# Members of the *Hyposoter didymator *Ichnovirus *repeat element *gene family are differentially expressed in *Spodoptera frugiperda*

**DOI:** 10.1186/1743-422X-3-48

**Published:** 2006-06-19

**Authors:** L Galibert, G Devauchelle, F Cousserans, J Rocher, P Cérutti, M Barat-Houari, P Fournier, AN Volkoff

**Affiliations:** 1 UMR1231 INRA-UMII Biologie Intégrative et Virologie des Insectes (BIVI), Place Eugène Bataillon, Case Courrier 101, 34 095 Montpellier Cédex5, France; 2 UMR 5160 CNRS-UMI Baculovirus et Thérapie, 30 380 Saint Christol-lez-Alès, France

## Abstract

**Background:**

The abundance and the conservation of the *repeated element *(*rep*) genes in Ichnoviruses genomes suggest that this gene family plays an important role in viral cycles. In the Ichnovirus associated with the wasp *Hyposoter didymator*, named HdIV, 10 *rep *genes were identified to date. In this work, we report a relative quantitative transcription study of these HdIV *rep *genes in several tissues of the lepidopteran host *Spodoptera frugiperda *as well as in the *H. didymator *wasps.

**Results:**

The data obtained in this work indicate that, in the early phases of infection (24 hours), HdIV *rep *genes each display different levels of transcripts in parasitized 2^nd ^instar or HdIV-injected last instar *S. frugiperda *larvae. Only one, *rep1*, is significantly transcribed in female wasps. Transcript levels of the HdIV *rep *genes were found as not correlated to their copy number in HdIV genome. Our results also show that HdIV *rep *genes display different tissue specificity, and that they are primarily transcribed in *S. frugiperda *fat body and cuticular epithelium.

**Conclusion:**

This work is the first quantitative analysis of transcription of the ichnovirus *rep *gene family, and the first investigation on a correlation between transcript levels and gene copy numbers in Ichnoviruses. Our data indicate that, despite similar gene copy numbers, not all the members of this gene family are significantly transcribed 24 hours after infection in lepidopteran larvae. Additionally, our data show that, as opposed to other described HdIV genes, *rep *genes are little transcribed in hemocytes, thus suggesting that they are not directly associated with the disruption of the immune response but rather involved in other physiological alterations of the infected lepidopteran larva.

## Background

Polydnaviruses are obligatory endosymbionts of some endoparasitic Hymenoptera from Ichneumonid and Braconid families. They are integrated as provirus in wasp chromosomes. Viral replication occurs in calyx cells of the wasp ovary, and leads to the formation of multiple circular dsDNA encapsidated molecules. Viral particles accumulate in the oviducts and are injected through oviposition in the lepidopteran host larva.

Polydnaviruses do not replicate in the parasitized lepidopteran host, but infect several host tissues, what leads to viral gene expression in these tissues. Polydnaviruses induce major physiological alterations in parasitized host such as immune disruption, developmental arrest, hormonal alterations and a decrease in hemolymph storage proteins [1-5].

Recent sequencing programs of the polydispersed polydnavirus genomes reveal that a large proportion of the genes encoded by the circular DNA molecules are organized in gene families. This characteristic is common to the two polydnavirus families, the Ichnoviruses (IV), associated with ichneumonid wasps, and the Bracoviruses (BV), associated with braconid wasps [6,7]. We are studying the Ichnovirus associated with the endoparasitoid wasp *Hyposoter didymator *(HdIV), where several gene families have been identified so far [7,8]. Although only a fraction of HdIV genome is presently known, 10 members of a gene family named *repeated element *(*rep*) gene family have already been identified in the genome. Originally described by Theilmann & Summers [9] on the basis of multiple cross-hybridization between several *Campoletis sonorensis *IV (CsIV) genome segments, members of the *rep *family possess a conserved 540-bp repeated element motif, found singly or in multiple repeats [9-12]. The *rep *gene family is the largest conserved ichnovirus gene family identified to date [7,13]. Indeed, 30 members of the *rep *gene family have been reported in the fully sequenced CsIV genome, whereas 36 and ten *rep *genes are described in *Hyposoter fugitivus *IV (HfIV) and *Tranosema rostrale *IV (TrIV), respectively [7].

Although the function of the *rep *genes has not yet been elucidated, their conservation among ichnoviruses and their abundance in viral genomes both suggest that they play an important role in viral cycles. To date, transcription studies for ichnoviruses *rep *genes have been carried out by Northern blot analysis [12,14] or by RT-PCR [11] and have indicated that members of this gene family may be transcribed in both wasp and caterpillar hosts [11,14] and in different tissues of the parasitized lepidopteran host [12,14]. Variations in the number of transcripts during the first day after parasitism have also been suggested for members of this gene family by Northern-blot analysis [14]. Altogether, these results seem to indicate that *rep *genes show a wide range of expression patterns, making it difficult to identify any putative physiological function. Based on the abundance of *rep *genes in ichnoviruses genomes, one might expect that they have diverged in their expression pattern, acquiring specificity for given tissues, hosts or development stages.

In this work, we report the relative quantitative transcription study of the 10 *rep *genes identified to date in HdIV. The transcription studies were carried out on several tissues of the lepidopteran host *Spodoptera frugiperda *as well as in *H. didymator *adult wasps. Our data indicate that 24 hours after infection HdIV *rep *genes display different levels of transcription in parasitized or HdIV-injected *S. frugiperda*. Surprisingly, one of the *rep *genes, *rep1*, is significantly transcribed in female wasps. However, *rep *genes remain preferentially transcribed in the lepidopteran host compared to the wasp host. Our data show that transcription levels of the HdIV *rep *genes are not correlated to their copy number in HdIV genome. In addition, each HdIV transcribed *rep *gene displays tissue specificity, and the primary targets are the lepidopteran host fat body and cuticular epithelium.

## Results and discussion

In HdIV, 10 *rep *genes are identified at present. Three have been previously described in HdIV segment SH-E (*rep1*, *rep2 *and *rep3*, [12]) and one in segment SH-G (named *rep12 *in this work, [15]). More recently, six additional sequences have been identified, which are available in the GenBank database (accession numbers in Table 4).

### Genome distribution and sequence analysis of HdIV *rep *genes

Characterisation of the segments containing the six new *rep *genes (*rep4*, *rep5*, *rep6*, *rep7*, *rep8 *and *rep11*) was achieved by Southern-blot analysis and PCR amplification of the corresponding circular molecules.

**Figure 1:**
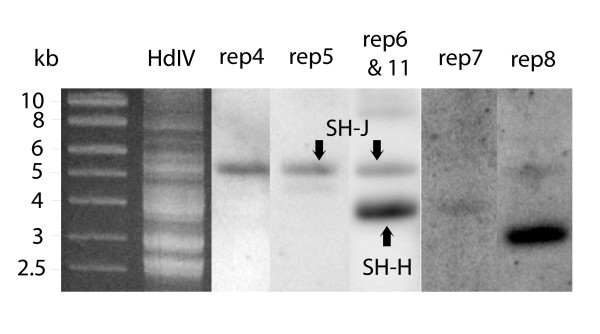
Characterization of the HdIV genomic segments encoding the novel 6 *rep *genes by Southern-blot analysis with gene-specific oligonucleotide probes. The molecular weight marker corresponds to linear DNA (kb). Purified HdIV DNA was separated on 1% agarose gel and stained with BET (HdIV), then transferred to Nylon membrane for hybridization with oligonucleotide probes specific to *rep4 *(rep4), *rep5 *(rep5), *rep7 *(rep7) and *rep8 *(rep8) genes. Due to high similarity between the *rep6 *and *rep11 *coding sequences, the *rep6 *probe (rep6&11) should allow detection of both genes. SH-J, containing *rep5 *and *rep11 *genes, and SH-H, containing the *rep6 *gene, are indicated by vertical arrows.

Southern-blot of HdIV segmented genome was performed with oligonucleotide probes specific to each of the newly identified *rep *genes, except for *rep6*. Indeed, *rep6 *and *rep11 *share 98 % nucleotide identity in their coding sequence and thus the *rep6 *probe was expected to cross-hybridize with *rep11*. As shown in Figure 1, the *rep4 *primer resulted in a hybridization band that co-localised with the hybridization band obtained with the *rep5 *primer (Figure 1, compare *rep4 *and *rep5 *lanes). The *rep6 *probe hybridized with two HdIV segments, firstly with a segment that co-localizes with the *rep5 *segment and secondly with another segment of lower size (Figure 1, *rep6 *lane). The faint hybridization band obtained with the *rep7 *probe had a size similar to that of the lower size segment to which the *rep6 *probe hybridized (Figure 1, compare *rep7 *and *rep6 *lanes). Lastly, the *rep8 *probe hybridized with a segment of smaller size compared to the other *rep *gene-containing HdIV segments. Thus, the Southern-blot analysis indicated that the 6 new *rep *genes are encoded by at least 3 different HdIV segments (Table 1).

**Table 1:** HdIV segments predicted to encode the *rep *genes analysed in this work. Segment names and putative sizes are indicated. Segment names were given alphabetically from the shortest to the longest; however only segments for which a real (completely sequenced; SH-E and SH-G) or estimated (PCR fragment; SH-J, SH-H and SH-A2 containing *rep7 *sequence) size could be given were named. SH-x and SH-y stand for segments of unknown size (since molecular weigh marker represents linear DNA).*Rep7 *is underlined because of discrepancy between PCR and Southern-blot results. For each segment, the *rep *gene(s) identified after sequencing of PCR amplification fragments or by Southern-blot analysis are reported

**segment**	**size (kbp)**	**sequencing/PCR**	**Southern-blot**
SH-J	~6	*rep5, rep11*	*rep4*
SH-y	~5	--------	*rep7?*
SH-H	~5	*rep6*	
SH-G	5.6	*rep12*	
SH-E	4.6	*rep1, rep2, rep3*	
SH-x	?	--------	*rep8*
SH-A2	3.1	*rep7*	

The HdIV *rep*-encoding segments were further analysed by PCR. Primers specific to the *rep5 *gene amplified a ~6 kbp fragment, whereas those designed within the *rep6 *gene amplified a ~5 kbp fragment. The HdIV super-helical (SH) segments are named alphabetically from the shortest to the longest. Thus, based on their size, the segment containing the *rep5 *gene was named SH-J and the one containing *rep6 *was named SH-H. Presence of the *rep5 *and *rep6 *genes was confirmed by partial sequencing of the two PCR fragments (GenBank accession numbers DQ295920 and DQ295919, for the segments SH-J and SH-H respectively). Sequencing revealed that SH-J also contained a sequence corresponding to the *rep11 *gene, thus confirming Southern-blot analysis where the *rep6 *probe hybridized with SH-H and cross-hybridized with *rep11 *present in SH-J (Figure 1, *rep6 *lane) whereas the *rep5 *probe hybridized solely with SH-J (Figure 1, compare *rep5 *and *rep6 *lanes). PCR using primers specific to the *rep7 *gene resulted in a 3.1 kbp fragment. Sequencing of this PCR fragment (GenBank accession number DQ295918) revealed a sequence identical to *rep7*. However, Southern-blot analysis suggested that a larger segment encodes this gene (Figure 1, *rep7 *lane). The discrepancy between the two results could be explained by the existence of two segments encoding this gene, similar to the *rep1 *gene, which is encoded by both SH-E and SH-Evar [12]. However, this result will need to be confirmed by identification and sequencing of the *rep7 *hybridizing segment.

Therefore, based on both Southern-blot and PCR results, we can conclude that the 10 HdIV *rep *genes are encoded by at least 5 different HdIV molecules (Table 1).

**Figure 2:**
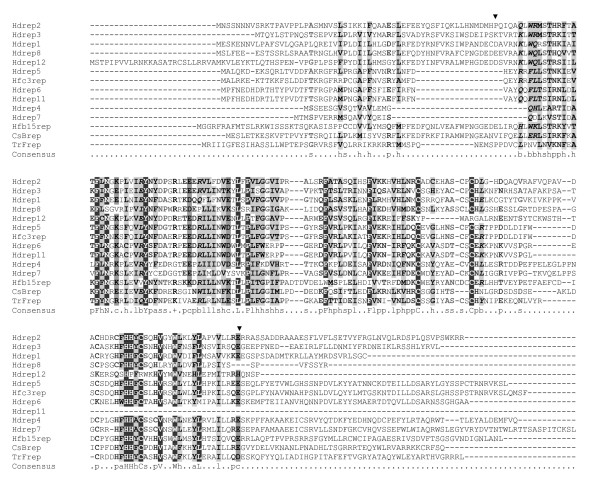
ClutalX alignment of deduced amino acid sequences of HdIV and selected ichnoviruses *rep *genes. The first 2 letters indicate ichnovirus species (Cs: *Campoletis sonorensis*; Hd: *Hyposoter didymator*; Hf: *Hyposoter fugitivus*; Tr: *Tranosema rostrale*), followed by the name of the segment containing the corresponding gene (except for Hd ichnovirus) then the *rep *gene number. Arrow-heads indicate beginning and ending of the conserved repeated element motif as defined by Theilmann & Summers [14]. Different shades of grey indicate conserved residues. Consensus sequence represents conserved residues: in capital letters: residues with >80% identity; p: polar residue; h: hydrophobic residue; l: aliphatic residue; +: positive residue; b: big residue; s: small residue.

Members of the *rep *family are characterized by a conserved 540-bp repeated element motif, found singly or in multiple repeats [9,11]. All the 10 *rep *genes identified to date in HdIV encode proteins containing a single repeated element motif (Figure 2). However, only part of the HdIV genome is presently known and therefore we cannot exclude existence of multiple-repeat containing genes in this Ichnovirus.

All the *rep *genes described to date lack intron and encode proteins with no predicted signal peptide [11,12,14]. The HdIV deduced rep proteins analysed in this work follow this rule. Moreover, immunofluorescence studies in cell lines transfected with rep proteins coupled in their C-terminal part to GFP confirm that the GFP-rep1, rep3 and rep5 proteins are intracellular (Galibert *et al*., unpub.).

ClustalX alignment [16] of rep proteins from HdIV and from other Ichnoviruses reveals a high degree of conservation in the repeated element motif (Figure 2). In contrast to the repeat element motif, the N-terminal and C-terminal sequences greatly diverge among the different rep sequences. The close similarity between rep6 and rep11 (at both nucleotide and amino acid levels) suggests the genes have diverged recently. Surprisingly, rep11 lacks the C-terminal part of the repeated element motif, compared to the other rep proteins.

Whole rep protein sequences of several Ichnoviruses containing a single repeat and accessible on GenBank database (10 HdIV, 2 HfIV, 1 TrIV and 25 CsIV proteins) were aligned by ClustalX [16] to generate trees (data not shown). Results did not indicate a clustering by virus species, regardless of the method used, distance and parsimony (PHYLIP package [17]), but rather a dispersion of HdIV sequences among the other ichnovirus sequences (data not shown). This distribution was different from that seen in previous studies, comparing a lower number of sequences, where rep proteins clustered by virus species [12]. Phylogenetic analysis of this important and diversified gene family would require supplementary studies, in order to understand if *rep *genes are derived from a single ancestor gene or if several *rep *genes existed prior to the association between an ichneumonid wasp and a polydnavirus ancestor.

### Transcription in the parasitized lepidopteran host

Transcription of the 10 HdIV *rep *genes was analysed by quantitative PCR during the first 24 hours following parasitism in *S. frugiperda *larvae parasitized at their 2^nd ^instar. Larvae parasitized by *H. didymator *rapidly exhibit reduced food consumption and growth, and their development is arrested at the end of the fourth larval instar, after the 8 days needed for completion of parasitoid larval development.

**Figure 3:**
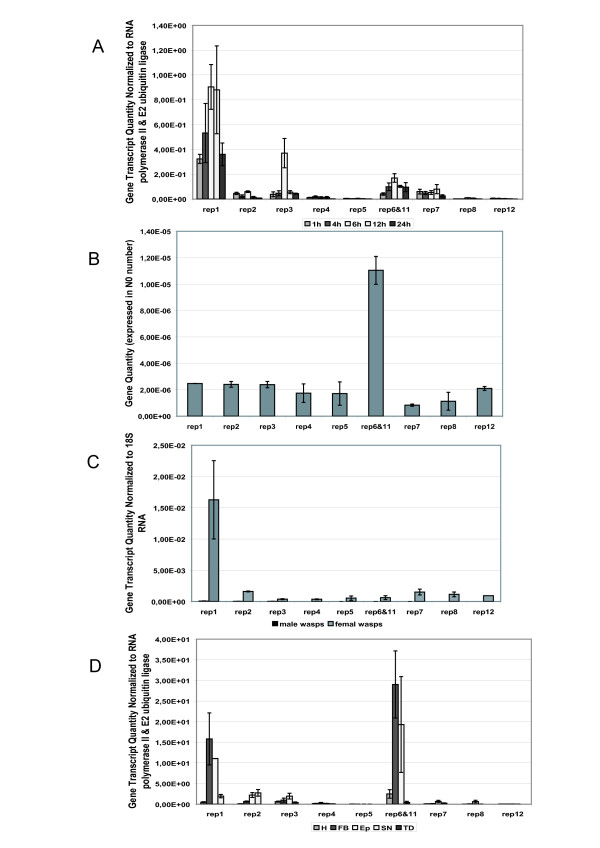
Expression profiles and gene copy number of the 10 *rep *genes identified in HdIV by relative quantitative PCR. **A**. Transcript levels in 2^nd ^instar *S. frugiperda *parasitized larva, over 1-h to 24-h time course study. **B**. Relative gene copy numbers in HdIV genome. **C**. Transcript levels in *H. didymator *adult female and male wasps. **D**. Transcript levels in different tissues of last instar *S. frugiperda *larvae 24 hours after injection of HdIV (H: Hemocyte; FB: Fat Body; Ep: Cuticular Epithelium; SN: Nervous System (Head); TD: Digestive Track). Data are means ± SE of starting quantity of fluorescence (N0 value) for 6–9 measurements. For **A**, **C **and D, data are normalized to housekeeping genes RNA polymerase II and E2 ubiquitin ligase. For details, address to Methods chapter.

Our data reveal that in the initial phases of parasitism important differences are found between the transcript levels of the different HdIV *rep *genes when considering the overall expression of the genes (Figure 3A). The highest level of transcripts corresponds to the *rep1 *gene, followed by the *rep7*, *rep3 *and *rep2 *genes. For example, 1 hour after parasitism, the ratios of *rep1 *transcripts (N0 value) to *rep3*, *rep2 *and *rep7 *transcripts are 8.4 ± 0.5, 7.0 ± 0.2 and 5.4 ± 0.3, respectively; 24 hours after parasitism, the ratios of *rep1 *compared to *rep3*, *rep2 *and *rep7 *are 8.2 ± 0.3, 43.3 ± 0.3, and 14.2 ± 0.3, respectively. Because of the high degree of identity between the *rep6 *and *rep11 *sequences, we were not able to design pairs of primers specific for each of the genes. Therefore, the results obtained in quantitative PCR include both genes. Nonetheless, *rep6 *and *rep11 *transcript levels are generally similar to *rep7 *and significantly higher than other *rep *genes such as *rep4*, *rep8*, *rep5 *or *rep12*. The *rep5 *and *rep12 *transcripts are detected at very low levels in the parasitized larvae (557.6 ± 0.3 fold and 1789.0 ± 0.3 fold respectively less than *rep1 *at 24 h post-parasitism) suggesting that transcription of these two genes in the lepidopteran host may have no real biological significance.

As indicated in Figure 3A, transcript levels remain relatively constant inside the whole parasitized *S. frugiperda *larvae over the first 24 hours of parasitism, for each of the HdIV *rep *genes with the exception of the *rep3 *gene, which appears to have transcript levels that are 6-fold higher at 6–9 hours post-parasitism compared to other points in the kinetic. These results are consistent with those obtained by Theilmann & Summers [14] in CsIV who observed through Northern blot experiments that some *rep *genes were slightly more transcribed 2 h and 6 h after parasitism than latter in parasitism (1d-8d). Nevertheless, the biological significance of this peak of transcription for the HdIV *rep3 *gene needs to be further investigated.

Transcript levels of the HdIV *rep *genes were also analysed during the time course of parasitism (data not shown). Preliminary results indicate that the differences in transcript levels between the HdIV genes are similar to those observed in early phase of parasitism. Moreover, transcript levels remain constant over the duration of parasitoid development. Therefore, the 10 HdIV *rep *genes studied here do not show variations in the course of parasitism as it has been described for some bracovirus genes [18].

In HdIV, the differences in transcript levels of the *rep *genes inside the whole parasitized *S. frugiperda *larvae are not related to their corresponding gene copy number in the HdIV segmented genome. Indeed, *rep1 *is more transcribed than both *rep2 *and *rep3 *although the 3 genes are found on the same viral segments SH-E and SH-Evar [12] and thus display the same gene copy numbers in the HdIV genome. Different patterns and levels of transcripts in the parasitized host for genes located on the same polydnavirus segment have also been previously described for the CsIV [19] and for the Bracovirus associated with *Chelonus inanitus *[18]. The absence of a correlation between transcript level and gene copy number was further assessed by estimating the relative copy numbers of each of the 10 *rep *genes from purified HdIV DNA (Figure 3B). As expected, our quantitative PCR assay revealed similar numbers of gene copies for the genes encoded by the same segments, *rep1*, *rep2 *and *rep3*. Furthermore, our results indicate that *rep1*, for which a high level of transcripts was detected, and *rep12*, a gene that is almost not transcribed, have similar numbers of copies within the HdIV genome (Figure 3B, compare rep1 and rep12). Overall, our data indicate that there are no significant differences within the HdIV genome between the copy number for *rep1*, *rep4*, *rep5*, *rep7*, *rep8 *and *rep12*. The *rep6 *and *rep11 *genes represent an exception (Figure 3B, rep6&11). Since *rep6 *and *rep11 *genes are both amplified by *rep6 *primers, the N0 value indicated in Figure 3B corresponds to the sum of *rep6 *(segment SH-H) and *rep11 *(segment SH-J) gene copies. The proportion of the N0 value due to *rep11 *can be estimated by the value obtained for *rep5*, since both genes are on the same segment SH-H. This indicates that *rep11 *gene (on SH-H segment) represents 15.5% of the total N0 value, whereas the *rep6 *gene (on SH-J segment) represents 84.5% of the N0 value. On the other side, quantification of the signal intensity obtained on Southern-blot (Figure 1, column rep6&11) indicates that SH-J (containing *rep6 *gene) and SH-H (containing *rep11 *gene) represent 78% and 15% hybridization signal, respectively. A third hybridization signal with a high molecular weight segment, representing 7% of the total signal intensity, was also detected in Southern-blot (Figure 1). Taken together, our results indicate that SH-J, containing the *rep6 *gene, is represented at least 5 times more than other *rep*-containing segments. Thus, although more abundant, *rep6 *is less transcribed than *rep1 *in parasitized larvae, result that confirms absence of correlation between copy numbers and transcription levels for the analysed HdIV *rep *genes.

To conclude, our results indicate that *rep *genes transcript levels are variable inside the parasitized caterpillars and are not linked to their relative copy numbers on HdIV genome thus suggesting that transcript levels of the HdIV *rep *genes are directly correlated to their promoter activities.

### Transcription in the wasp host

Since some of the CsIV *rep *genes are transcribed in both lepidopteran and hymenopteran hosts [11,14], we investigated transcription of HdIV *rep *genes in 2–3 days old *H. didymator *female and male adult wasps. At this time, viral replication is taking place in the calyx cells [20]. In the female wasps, the *rep *genes are transcribed, but at a very low level, with the exception of *rep1*, which was significantly more transcribed compared to the other *rep *genes (Figure 3C). In the male wasps, transcript level is more than 200-fold lower than in females, suggesting that transcription of HdIV *rep *genes is residual in male wasps. This result differs from previous reports on *C. inanitus *bracovirus where 5 out of 6 analysed CiBV genes were transcribed at similar levels in male and female wasps [18]. The finding that transcription of *rep1 *gene is restricted to *H. didymator *females suggests an unexpected complex regulation of gene transcription, regardless transcripts are generated from the integrated or from the excised viral DNA. The remaining question is if *rep1 *transcription is restricted or not to the replicative calyx cells and thus if it may be related to HdIV viral particle production.

The HdIV *rep1 *gene is therefore the most transcribed *rep *gene in both parasitized *S. frugiperda *and whole adult female wasps. Whether transcription of *rep1 *gene is more important in parasitized *S. frugiperda *larvae than in the female wasp remains to be clearly established. By assuming that reverse transcription and PCR efficiencies were identical in the samples issued from both *S. frugiperda *larvae and female wasps, we were able to compare the N0 values obtained. In both samples, non-normalized N0 values are around 4E-07, which indicates that *rep1 *transcript levels are similar in both insect hosts. We can therefore assume that transcription of the *rep1 *gene in female wasps has a biological significance although it remains to be clarified whether the related protein has a function in the wasp and if this function is the same as that in the parasitized lepidopteran host.

### Pattern of transcription in different tissues of HdIV-infected *S. frugiperda *larvae

In order to assess if HdIV *rep *genes have tissue specific patterns of transcription, quantitative analysis was performed in different tissues of *S. frugiperda *last instar larvae. Our results show that, 24 hours after HdIV injection, the HdIV *rep *genes are preferentially transcribed in the fat body and cuticular epithelium, and to a lower extent in the nervous system of the infected host (Figure 3D). Finding of a preferential transcription of the *rep *genes within these 3 tissues is consistent with previous results obtained by Northern-blot analysis for the HdIV *rep1 *gene [12].

In HdIV-injected last instar larvae, as in the parasitized 2^nd ^instar larvae, *rep4*, *rep5*, *rep8*, *rep12*, but also *rep7 *show very low transcript levels in all tissues examined, whereas *rep1*, *rep6*, and to a lower extent, *rep2 *and *rep3*, are detected at higher levels (Figure 3D). In this assay, where tissues are analysed individually, *rep1 *transcripts are not any longer the most abundant. Indeed, *rep6 *transcripts level is similar to that of *rep1 *transcripts, in particular in the fat body and cuticular epithelium (despite a high variation between the biological samples for *rep6 *in cuticular epithelium, as indicated by the standard error, Figure 3D). This result has to be modulated by the fact that, in this assay, both *rep6 *and *rep11 *transcripts were measured and the proportion of each of the two genes is not known. However, *rep6 *preferential transcription in HdIV-injected last instar larvae fat body was corroborated by Northern blot analysis using *rep *genes specific oligonucleotide probes. Indeed, only one hybridization signal was detected, which corresponded to the *rep6 *specific probe in the fat body tissue (data not shown).

Our results indicate that the highest levels of *rep *gene transcripts are detected in the fat body and the cuticular epithelium (Figure 3D). Other ichnovirus genes of unknown function, such as TrIV1, also target primarily the fat body and the cuticular epithelium, with few transcripts detected in hemocytes [21]. In these two tissues, the HdIV *rep6 *and *rep1 *are the most represented transcripts, both at comparable levels. The *rep2 *and *rep3 *transcripts are also detected in fat body and cuticular epithelium, but at levels approximately 10 to 15-fold lower than those of *rep1 *and *rep6 *genes. In the nervous system, we detected mainly *rep2 *and *rep1 *transcripts, although at lower levels than in fat body and cuticular epithelium. For example, *rep2*, the highest transcribed gene in the nervous system, has 5-fold fewer transcripts than the *rep1 *gene in fat body. Compared to others tissues, transcripts in the digestive tract are almost undetectable for all the genes considered, suggesting that *rep *genes do not target this tissue. Whether this is due to promoter activity or virus penetration in this tissue remains to be determined.

Injection of purified viral HdIV particles inside *S. frugiperda *last instar larvae induces the inhibition of the cellular immune response and results in reduction of larval growth leading to abnormal or lack of pupation. Interestingly, the *rep *genes are expressed at low levels in hemocytes, as opposed to other HdIV genes [8,15,22] or genes from other polydnaviruses, which frequently preferentially target the blood cells [18,19,23,24]. The only *rep *gene that is transcribed significantly in the hemocytes is *rep6 *but transcript levels are still 6-fold less than in fat body and cuticular epithelium.

Based on the nature of the tissues where HdIV *rep *genes are preferentially transcribed and on the fact that rep proteins remain intracellular, we can hypothesize that members of this gene family play a small or an indirect role in cellular immune-suppression. The *rep *genes may thus mediate other physiological alterations of the parasitized caterpillar such as developmental/growth arrest.

## Conclusion

This study by relative quantitative PCR allowed us to demonstrate that a number of HdIV *rep *genes are not transcribed at the same levels in the parasitized lepidopteran host. Even if transcript levels do not account for protein activity and needs, we can make hypotheses to explain the low transcript levels seen for some of the *rep *genes (*rep4*, *rep5*, *rep8*, *rep12*). Firstly, *rep *genes could be involved in host range for *H. didymator *wasp and those genes could be more transcribed inside other hosts. Another possibility is that these low transcribed *rep *genes have become pseudogenes, through genomic rearrangement in the wasp DNA. For example HdIV SH-G contains *rep12 *and *HdGorf1*, but the two open reading frames are on complementary strands [15]. Differences in transcript level between *HdGorf1*, which are similar to those of *rep1 *(data not shown), and *rep12 *could be related to their orientation on the viral segment. A third possibility would be that the *rep *genes that were not detected in fat body, cuticular epithelium or nervous system are expressed in other, less abundant tissues such as the endocrine glands.

HdIV *rep *genes seem to be specifically transcribed into the Lepidoptera host rather than in the Hymenoptera host, except maybe for the *rep1 *gene. In infected *S. frugiperda *larvae, the *rep *genes transcripts are detected mostly in fat body, cuticular epithelium and nervous system. Interestingly *rep3 *gene transcripts are found at the same level than *rep1 *transcripts in Sf9 cells infected with HdIV (data not shown), showing that viral gene regulation can differ in *in vivo *and *in vitro *systems.

The question whether *rep *genes have the same functions in different tissues has yet to be answered. Based on their transcription profiles, it is possible that *rep *genes do not have a direct role in the disruption of the immune response of the infected lepidopteran larva, but rather that they contribute to the manipulation of lepidopteran host larval growth and development.

## Methods

### Insect material

Rearing of *Spodoptera frugiperda *larvae and *Hyposoter didymator *wasps, as well as HdIV virus and DNA purifications, were conducted as described in [8].

For transcriptional studies in parasitized *S. frugiperda *larvae, second instar larvae were placed in presence of *H. didymator *female wasps for 3 hours. Negative controls corresponded to non-parasitized larvae.

To study the transcription of HdIV *rep *genes in HdIV-injected *S. frugiperda *larvae tissues, purified virions were injected into *S. frugiperda *last instar larvae (3 wasps equivalent/larva, representing 28 μl). For negative controls, last instar larvae were injected with an identical volume of saline buffer (PBS).

### Southern blot analysis for identification of HdIV *rep *genes

Identification of HdIV segments containing the new *rep *genes was carried out by Southern blot analysis. 3 μg of purified HdIV DNA and linear DNA molecular weight marker (Eurogentec) were migrated on 1% agarose gel and transferred on positively charged nylon membranes (Boehringer). Gene specific oligonucleotide probes were selected in the coding sequence of each *rep *gene (sequences in Table 2). Specificity of the probe was ascertained with Blastn at the NCBI . Membranes were pre-hybridized for 3 hours at the same temperature than hybridization (see below) in a solution containing 5X Denhardt, 5X SSC, 0.1% SDS, and 100 μg/ml of salmon sperm DNA. Hybridization was carried out for 20 hours with oligonucleotide probe specific of each *rep *gene (hybridization temperature is indicated next to the primer in Table 2). The probes were labelled using γ-^32^P-ATP with T4 polynucleotide kinase (Promega). A DNA weight marker was hybridized with linear pUC-18 DNA labelled with α-^32^P-dCTP in a random priming reaction in the same conditions as described above (hybridization temperature 42°C). Membranes were rinsed at room temperature twice for 5 minutes in 2X SSC; 0.1% SDS solution, and once for 10 minutes in 0.2X SSC; 0.1% SDS solution. PhosphorImaging was performed on a STORM 840 apparatus (Amersham). Quantification of bands intensity was performed using ImageQuant 5.2 software from Amersham.

**Table 2:** List of the gene-specific oligonucleotide probes used in Southern-blot. Hybridization temperature is indicated next to the primer

**Gene**	**oligonucleotide (5'-3')**	**Pre-hybridization & hybridization temperature**
*rep4*	GATGTTGCCCCATTTCTAGAACCGCAACAG	48°C
*rep5*	AGGGGCCCCACGCGGTAGACGAAACCCACG	54°C
*rep6 *&*11*	GCCCGCGGAACGTGAAGGTGTCCACCGGGT	50°C
*rep7*	CCTGCGAAATTTCTTGATACACCACAGCCT	47°C
*rep8*	TTCTCGTTGCAGCCCGTGACAGGCGCGAGC	53°C

### PCR amplification of HdIV *rep*-containing segments

Characterization of HdIV segments containing the new *rep *genes was conducted by PCR with primers specific for each *rep *gene (Table 3). PCR was conducted with High Fidelity Taq DNA polymerase (Invitrogen) in standard conditions with 0.1 μg of DNA as a template and an annealing temperature of 60°C.

**Table 3:** List of primers used to amplify the *rep*-containing HdIV segments by Polymerase Chain Reaction

**HdIV segment**	***rep *gene**	**forward primer (5'-3')**	**reverse primer (5'-3')**
SH-A2	*rep7*	ATCTTAAAGTGAGCACTATTGACGC	CCTGCGAAATTTCTTGATACACCACAGCCT
SH-H	*rep6*	AAGTGTTGCTTGACTCGGCT	TCAAGTCCAGGTTTCGGATC
SH-J	*rep5*	CTTGGTTACTCCAGCCCTTG	ACTCCTCCGAATAAAGGCGT

**Table 4:** List of the gene-specific primers used in relative quantitative PCR analysis. Gene names and accession numbers are indicated. (*) Accession numbers for *S. frugiperda *correspond to Spodobase identifying numbers

**gene name**	**accession number**	**qPCR forward primer (5'-3')**	**qPCR reverse primer (5'-3')**
**HdIV**			
*rep1*	AF364055	AACGTGGAAACTTTGTCGCC	CGTTCCTGGAGGGACTACCC
*rep2*	AF364055	TCGGTGTGCTGATTGTGAGC	TCATGTCCCAAGTCACACGG
*rep3*	AF364055	GCCCCTGCCATTTGAAAAAT	TCGCGAATGCAGTAGCACTG
*rep4*	AY499565	CGGCGTGTCACAAACTGTTG	GCTTCAAGATGTTGCCCCATT
*rep5*	AY499566	GGAAGACCGCCTGCTTATCA	CCTCCGAATAAAGGCGTCAGT
*rep6*	AY499567	AAGGCCAGAAGAAGATCGCC	AGAGGCATGAGCCAGTCCC
*rep7*	AY499568	TCGTATCGTTCCACCGGGTA	CAGCCAGATGGTGGAAGCTC
*rep8*	AY499569	GTTTTGCCCCAATGGTGATG	TGCCACAGTTTTGCTCGAAC
*rep11*	AY501383	same as *rep6 *gene	same as *rep6 *gene
*rep12*	AF479654	GGGTCGCAATGAAGGTGCTA	CTGGCGAGTGTGTTTGCAAT
***H. didymator***			
18S RNA	AY433942	CATCGTGGTGCTCTTCATTGA	CAAAGTAAACGTACCGGCCC
***S. frugiperda***			
E2 ubiquitin ligase	SF9L03548 (*)	ACTTGTGGCCCGCATACACT	GGATCGGCACAATAAATGGG
RNA polymerase II	SF9L00930 (*)	TGCCATCGGGAAAATGAAAT	TTCTCTGCACCTTATTGGGTCTC

### Sequence analysis

Alignment of the deduced amino acid sequences encoded by the 10 HdIV *rep *genes, the HfIV *rep *genes (*Hfc3rep *(GenBank: AY597815) and *Hfb15 rep *(GenBank: AY570798)), the TrIV *TrFrep *gene (GenBank: AF421353) and the CsIV *CsBrep *gene (GenBank: AAA42923) was carried out with ClustalX [16] using default settings.

### RNA isolation

To study the transcript levels of the *rep *genes in parasitized *S. frugiperda *larvae, total RNA was isolated from second instar larvae 1 h, 4 h, 6 h, 12 h and 24 h after parasitism. For each time point, 15 larvae were collected and homogenized in 1 mL TRIzol reagent (Invitrogen). For tissue specific transcription analysis, tissues were collected from 10 last instar HdIV-injected *S. frugiperda *larvae, 24 h after injection of HdIV or PBS. The tissues collected were hemocytes, digestive track, head (for nervous system), fat body and cuticular epithelium (including the muscles attached to the cuticle). With the exception of hemocytes, which were directly collected in TRIzol reagent, tissues samples were rinsed in PBS prior to collection. Tissues were then ground in 1 mL of TRIzol reagent. For the wasps' samples, 2 days old female and male wasps (20 of each) were ground in 1 mL TRIzol reagent. For each assay, RNA was collected from three independent sets of insects (biological replicates).

Total RNAs were extracted following the manufacturer's protocol. Total RNA samples were then incubated overnight at -20°C in 2 M of LiCl, centrifuged 30 min at 7500 g, rinsed 2 times with ethanol 75% and re-suspended in nuclease free water (Promega). RNA samples were quantified through spectrometry. The quality of the extracted RNA was confirmed on a 1% agarose gel.

To eliminate contaminating DNA, 8 μg of each RNA sample were treated with 8U of RQ1 DNAse (Promega) for 3 h at 37°C, following the manufacturer protocol. Samples were then ethanol precipitated with sodium acetate, rinsed twice in 75% ethanol and re-suspended in nuclease free water. The RNA samples treated with RQ1 DNAse were checked by PCR for the absence of contaminating DNA before being submitted to RT-PCR. For the *S. frugiperda *RNA samples, the absence of genomic contaminating DNA was controlled with primers amplifying the actin sequence (forward 5'-CAACTGGGACGACATGGAGAAGAT-3'; reverse 5'-CCACCGATCCATACGGAGTATTTC-3'). The absence of viral DNA contamination was controlled with primers amplifying the sequence of HdIV *rep6 *gene (forward 5'-ATGCCGTTCAACGAAGATCACGAC-3'; reverse 5'-GCTGCACCATGGCCGGAACTG-3'). For the *H. didymator *RNA samples, we used the primers amplifying the *rep6 *gene to control both absence of wasp and viral genomic DNA. The following protocol was used: 0.5 μg of each RNA sample served as matrix for RT-PCR using SuperScript™ III One-Step RT-PCR System with Platinum Taq DNA Polymerase (Invitrogen) and PCR using Platinium Taq DNA polymerase with the same buffer as the RT-PCR kit. The PCR program for both PCR and RT-PCR was 48°C 30 min; 94°C 5 min and 30 cycles 94°C 30 s; 55°C 30 sec; 1 min 68°C.

### cDNA synthesis for relative quantitative PCR

Reverse transcription was carried out on 8 μg of total RNA using SuperscriptII reverse transcriptase (Invitrogen), following the manufacturer's protocol. 1U of RNAsin plus (Promega) was added in the reaction medium. Reverse transcription was carried out for 3 h.

### Relative quantitative PCR

For Relative Quantitative PCR, primers were designed with the Primer Express software (version 2, Applied Biosystem). The gene specificity of the primers was verified using BLASTn (NCBI). The list of primers used is shown in Table 4. The primers for *rep1*, *rep2 *and *rep3 *were designed in such a way that the genes encoded by both SH-E and SH-Evar [12] were amplified.

For transcription studies, each of the 3 biological replicate samples was analysed in triplicate and non-template controls were included in duplicate or triplicate in each assay. Reactions were performed in 96-well PCR plates (ABgene). For PCR using HdIV DNA as template, 0.16 ng of DNA was used, and the experiment was conducted on two sets of independently collected DNA samples (biological replicates). For the PCR using cDNA as template, an amount of cDNA corresponding to 100 ng reverse transcribed total RNA was used. Each template was amplified in a volume of 25 μl containing 1X PCR buffer (Invitrogen), 3 mM of MgCl2, 200 μM dNTP mix (Invitrogen), 0.2 μl of 1/2000 dilution stock solution of SYBR green I (Invitrogen), 0.5 μM of ROX dye (Interchim), 0.4 μM of couples of primers and 0.1U of Platinium Taq DNA polymerase (Invitrogen).

Relative Quantitative PCR were performed on an ABI PRISM 7000 apparatus (Applied Biosystems) using the following thermal profile: 95°C 2 min and 40 cycles: 95°C 15 sec, 60°C 1 min. The specificity of the amplicons synthesised during the PCR was ascertained by performing a dissociation curve protocol from 60°C to 95°C.

### Relative quantitative PCR results analysis

Analysis of Relative Quantitative PCR results was performed with the program LinReg PCR developed by Ramakers *et al*. [25], using the Rn values (SYBR green I fluorescence normalized to ROX passive dye fluorescence, given by the Sequence Detection Software of Applied Biosystem) as entries. This approach gives the initial number of molecules presents in each sample (N0 value). The mean of the 3 technical replicates N0 values was calculated.

Transcription results, obtained in *S. frugiperda *larvae, were first normalized, according to Vandesompele *et al*. [26], to the geometrical mean of 2 selected housekeeping genes: the RNA polymerase II and the E2 ubiquitin-conjugating enzyme. These two genes were chosen because their ratio was constant regardless of the tissue studied. Transcription results obtained in *H. didymator *wasps were first normalized to the 18S RNA gene.

For comparison between biological replicates, we introduced a second normalization step, aimed at reducing variability due to possible different quantities of virus inoculated or parasitism rates. Using the geNorm program [26], we first controlled that, for a same tissue or for a same time in the kinetic study, each *rep *gene behaves similarly in the 3 biological samples. Then using the geNorm program, a normalization factor was calculated for each tissue or time point, taking the most stable genes identified by the previous control, with the M value (internal gene-stability measure) set to 3. After normalisation, average values and standard errors were calculated for the 3 biological replicates.

Normalisation of the *rep *gene copy numbers on HdIV genome between the 2 biological samples was carried out using geNorm program as described above.

## Abbreviations

HdIV: *Hyposoter didymator *IchnoVirus

PCR: Polymerase Chain Reaction.

*rep*: *repeat element *gene

## Competing interests

The author(s) declare that they have no competing interests.

## Authors' contributions

L. Galibert conducted the experiments, J. Rocher was in charge of amplifying *rep*-containing HdIV segments, F. Cousserans and M. Barat-Houari helped with qPCR experiments, G. Devauchelle and P. Cerutti identified the novel *rep *genes in HdIV genome, P. Fournier assisted in manuscript composition with A.-N. Volkoff.
